# Applications of Nanomaterials in Biomedical Imaging and Cancer Therapy: 3rd Edition

**DOI:** 10.3390/nano15231761

**Published:** 2025-11-24

**Authors:** James C. L. Chow

**Affiliations:** 1 Radiation Medicine Program, Princess Margaret Cancer Centre, University Health Network, Toronto, ON M5G 1X6, Canada; james.chow@uhn.ca; 2 Department of Radiation Oncology, University of Toronto, Toronto, ON M5T 1P5, Canada; 3 Department of Materials Science and Engineering, University of Toronto, Toronto, ON M5S 3E4, Canada

Following the success of our first two editions of “Applications of Nanomaterials in Biomedical Imaging and Cancer Therapy”, this third edition continues to highlight the rapid evolution of nanotechnology and its transformative impact on biomedical imaging, targeted therapy, and cancer theranostics [1,2]. The series has consistently provided a forum for researchers worldwide to showcase innovative nanomaterial-based approaches addressing some of oncology’s greatest challenges—treatment selectivity, biocompatibility, and clinical translation.

Building upon the foundations laid in the first and second edition [3,4], which emphasized nanoparticle-based imaging modalities, multifunctional composites, and radiation dose enhancement, this new collection extends the discussion to emerging trends in tumor microenvironment modulation, responsive nanocarriers, and computational modeling in nanomedicine. Collectively, the ten contributions in this issue present significant advancements that continue to drive the field toward precision, safety, and clinical readiness.

For innovative nanomaterials in cancer therapy and imaging, Chkair et al. [5] report the development of hydrophilic, fluorescent organic nanoparticles (FONPs) functionalized with purpurin-18 for photodynamic therapy (PDT) in colorectal cancer. Their study demonstrates efficient induction of apoptosis via reactive oxygen species (ROS) generation, highlighting a promising approach for minimally invasive treatment. In a complementary approach, Snyder et al. [6] explore the synergistic use of silver nanoparticles (AgNPs) with proteotoxic stress inhibitors to selectively induce death in triple-negative breast cancer (TNBC) cells. This work highlights how stress pathway modulation can refine nanotoxicity into a therapeutic advantage. The paper by Kitamura et al. [7] introduces a tumor microenvironment-responsive liposomal system incorporating iRGD-conjugated peptides to enhance tissue penetration. These nanoparticles exhibited Neuropilin-1-mediated tumor targeting, representing a promising advancement for siRNA and drug delivery deep within tumors. Cakir et al. [8] design chrysin-loaded micelles synthesized via RAFT polymerization, enabling dual apoptotic pathway activation in ovarian cancer cells. This multi-functional system combines hydrophobic drug encapsulation with precise endosomal escape and improved biocompatibility.

Focusing on nanoparticle engineering and mechanistic insights, Quispe Cohaila et al. [9] describe a green synthesis of biogenic zinc oxide (ZnO) nanoparticles using Bacillus licheniformis. These nanoparticles exhibit selective cytotoxicity against glioblastoma cells while maintaining normal retinal cell viability. Their findings highlight the promise of microbial nanomanufacturing as a safe and effective strategy for anticancer agent development. Zimina et al. [10] investigate the magnetically controlled transport of nanoparticles using a tumor-on-a-chip model. Their findings reveal how particle size and coating influence migration dynamics, offering insights for controlled nanoparticle delivery in dense tumor tissues. Moreover, the use of carbon-encapsulated iron nanoparticles for molecular MRI imaging of glioma, developed by Stawarska et al. [11], demonstrates targeted imaging through integrin αvβ3 receptors. This study contributes to non-invasive diagnosis and image-guided therapy development.

In the realm of modeling, simulation, and quantum-based nanomedicine, Monte Carlo modeling remains a powerful computational tool. FLASH radiotherapy (FLASH-RT) is an emerging modality that delivers radiation at ultrahigh dose rates (UHDRs), typically greater than 40 Gy per second. This technique has attracted significant attention for its ability to spare normal tissues while maintaining or even enhancing tumor control [12]. Chow [13] offers a comprehensive review of its applications in nanoparticle-enhanced radiotherapy, nanodosimetry, and the optimization of FLASH-RT. This review bridges computational and clinical nanoscience, emphasizing patient-specific and quantum modeling strategies for next-generation treatment planning. Kim and Chow [14] further explore this frontier by quantifying ROS yields around gold nanoparticles under UHDR electron beams. Their study elucidates nanoparticle–radiation interactions at the nanoscale, providing a computational foundation for combining gold nanoparticles with FLASH-RT. Moreover, Sarwat et al. [15] extend nanotechnology applications to ophthalmology through the development of hydrophobic silicon quantum dots (Si-QDs) for imaging the tear film lipid layer. This innovation exemplifies the versatility of nanomaterials beyond oncology, addressing the imaging challenges in dry eye disease.

The papers featured in this third edition collectively advance our understanding of how nanomaterials can revolutionize both diagnostic and therapeutic modalities. From photodynamic and radiotherapy enhancement to computational simulation and bio-inspired synthesis, these studies showcase the interdisciplinary depth and translational potential of nanotechnology in medicine.

Together with the first and second editions, this ongoing series emphasizes a central theme: the convergence of nanotechnology, biology, and computation is pivotal to advancing precision oncology. As emerging platforms such as tumor microenvironment-responsive nanoparticles, FLASH-RT optimization, and machine learning-driven nanomaterial design continue to mature, the boundary between simulation and clinical application grows increasingly narrow.

We thank all authors, reviewers, and collaborators for their exceptional contributions that make this Special Issue a vibrant reflection of nanomedicine’s promise.

## Data Availability

Not applicable.

## Abbreviations

The following abbreviations are used in this manuscript:

FONPs	Fluorescent Organic Nanoparticles
PDT	Photodynamic Therapy
ROS	Reactive Oxygen Species
AgNPs	Silver Nanoparticles
TNBC	Triple-Negative Breast Cancer
iRGD	Internalizing Arginine–Glycine–Aspartic Peptide
siRNA	Small Interfering RNA
RAFT	Reversible Addition–Fragmentation Chain Transfer
ZnO	Zinc Oxide
MRI	Magnetic Resonance Imaging
FLASH-RT	FLASH Radiotherapy
UHDR	Ultrahigh Dose Rate
Si-ODs	Silicon Quantum Dots
